# Supplementary material for: Development and Implementation of a Quadruple RT-qPCR Method for the Identification of Porcine Reproductive and Respiratory Syndrome Virus Strains
Source: Viruses. 2023 Sep 18;15(9):1946. doi: 10.3390/v15091946 (PMC10536281; doi:10.3390/v15091946)
Supplement: Supplementary file 1 [file viruses-15-01946-s001.zip › viruses-2546231-supplementary.pdf]

Supplementary Materials

Table S1. Optimal annealing temperature for single-plex quantitative fluorescence

Annealing temperature (°C)	FAM	ROX	HEX	CY5
60	25.33 ± 1.32	27.75 ± 0.67	23.80 ± 0.73	20.47 ± 0.66
59	24.16 ± 0.53	27.59 ± 0.22	23.71 ± 1.42	19.72 ± 1.73
58	24.82 ± 0.20	26.07 ± 0.31	23.28 ± 0.91	20.19 ± 1.05
57	24.16 ± 1.14	25.27 ± 1.58	23.13 ± 0.47	19.84 ± 1.52
56	25.16 ± 1.02	26.63 ± 1.40	22.72 ± 1.84	20.24 ± 2.04
55	24.32 ± 2.17	26.7 ± 2.28	23.05 ± 0.46	20.01 ± 2.55
54	24.45 ± 0.46	26.58 ± 1.12	23.04 ± 0.37	20.06 ± 1.20
53	24.5 ± 1.19	27.87 ± 0.69	22.86 ± 1.75	20.77 ± 0.92

Table S2. Optimal annealing temperature for the four virus plasmids was determined to be 59 °C

Annealing temperature (°C)	FAM	ROX	HEX	CY5
60	22.67 ± 1.72	24.49 ± 0.96	24.64 ± 1.56	24.33 ± 1.97
59	20.67 ± 1.38	23.73 ± 0.68	22.81 ± 1.27	22.85 ± 1.22
58	22.54 ± 0.82	24.13 ± 1.34	24.08 ± 1.04	23.78 ± 0.87
57	20.77 ± 2.41	23.81 ± 1.72	23.14 ± 0.98	22.93 ± 1.62
56	21.77 ± 1.88	23.92 ± 0.82	23.79 ± 1.62	23.54 ± 1.86
55	21.84 ± 2.07	23.93 ± 1.57	22.94 ± 1.59	23.46 ± 1.62
54	21.65 ± 1.36	23.5 ± 1.65	23.23 ± 2.42	22.99 ± 0.96
53	21.75 ± 1.81	24.1 ± 1.02	23.78 ± 1.68	23.25 ± 1.93

Table S3. Amplification results from concentration crossover experiment of primers and probes for PRRSV1-ORF7 viral plasmid

FAM (CT $\bar{x} \pm SD$)		Probes (μmol/L)					
		**0.2**	**0.3**	**0.4**	**0.5**	**0.6**	**0.7**
Primers (μmol/L)	**0.2**	21.14 ± 0.90	20.51 ± 1.56	20.3 ± 2.35	20.74 ± 2.33	20.86 ± 0.73	20.74 ± 1.57
	**0.3**	20.82 ± 1.67	20.72 ± 0.89	20.72 ± 1.67	20.33 ± 0.63	20.39 ± 1.25	20.37 ± 2.04
	**0.4**	20.84 ± 2.23	20.08 ± 1.59	20.64 ± 1.72	20.17 ± 1.55	20.74 ± 1.20	20.4 ± 1.35
	**0.5**	21.06 ± 1.82	20.9 ± 1.68	20.85 ± 1.46	20.87 ± 0.86	20.39 ± 1.39	20.76 ± 1.94
	**0.6**	21.16 ± 1.16	20.91 ± 2.46	20.15 ± 0.97	20.76 ± 1.54	20.62 ± 1.04	20.42 ± 2.26
	**0.7**	21.94 ± 0.68	20.42 ± 1.59	21.0 ± 1.45	20.71 ± 2.42	20.65 ± 1.65	20.37 ± 1.89

Table S4. Amplification results from concentration crossover experiment of primers and probes for PRRSV2-ORF7 viral plasmid

ROX (CT $\bar{x} \pm SD$)		Probes (μmol/L)					
		**0.2**	**0.3**	**0.4**	**0.5**	**0.6**	**0.7**
Primers (μmol/L)	**0.2**	19.66 ± 1.73	19.23 ± 1.11	18.51 ± 1.08	18.35 ± 1.68	18.37 ± 1.95	18.34 ± 1.68
	**0.3**	19.7 ± 1.87	19.45 ± 1.65	18.98 ± 1.53	18.6 ± 1.05	18.37 ± 1.84	18.46 ± 1.79
	**0.4**	19.77 ± 1.25	18.13 ± 1.86	18.41 ± 1.37	18.5 ± 1.64	18.53 ± 1.73	18.23 ± 1.18

0.5	20.03 ± 1.26	19.47 ± 1.23	19.18 ± 1.49	18.84 ± 1.80	18.52 ± 1.37	18.57 ± 1.30
0.6	19.97 ± 1.80	19.69 ± 1.87	19.34 ± 1.80	18.95 ± 1.63	18.73 ± 1.08	18.81 ± 1.03
0.7	21.12 ± 1.34	19.43 ± 1.39	19.35 ± 1.24	18.97 ± 1.43	18.77 ± 1.35	18.55 ± 1.64

Table S5. Amplification results from concentration crossover experiment of primers and probes for HP-PRRSV2-NSP2 viral plasmid

HEX (CT $\bar{x} \pm SD$)		Probes (μmol/L)					
		**0.2**	**0.3**	**0.4**	**0.5**	**0.6**	**0.7**
Primers (μmol/L)	**0.2**	24.3 ± 1.46	24.12 ± 0.75	25.34 ± 0.87	25.27 ± 1.53	25.5 ± 1.32	25.15 ± 1.26
	**0.3**	24.27 ± 2.08	24.13 ± 2.34	25.14 ± 2.26	25.14 ± 0.24	25.4 ± 2.12	24.99 ± 0.79
	**0.4**	24.52 ± 0.98	24.02 ± 1.18	25.16 ± 2.06	25.38 ± 0.89	25.69 ± 2.40	25.26 ± 1.14
	**0.5**	24.55 ± 1.61	24.25 ± 1.34	25.24 ± 2.05	25.15 ± 1.42	24.78 ± 1.84	25.39 ± 1.48
	**0.6**	24.47 ± 0.27	24.07 ± 0.79	25.16 ± 0.42	25.53 ± 1.86	25.1 ± 1.62	25.41 ± 0.73
	**0.7**	24.03 ± 0.84	24.98 ± 1.54	25.07 ± 1.35	24.79 ± 1.23	25.17 ± 0.98	24.14 ± 1.43

Table S6. Amplification results from concentration crossover experiment of primers and probes for C-PRRSV2-NSP2 viral plasmid

CY5 (CT $\bar{x} \pm SD$)		Probes (μmol/L)					
		**0.2**	**0.3**	**0.4**	**0.5**	**0.6**	**0.7**
Primers (μmol/L)	**0.2**	29.36 ± 0.84	30.22 ± 2.04	30.09 ± 1.97	30.03 ± 0.76	29.94 ± 0.79	29.67 ± 2.36
	**0.3**	29.7 ± 1.51	29.77 ± 1.63	29.99 ± 2.06	30.01 ± 0.83	30.04 ± 1.83	29.5 ± 2.44
	**0.4**	29.81 ± 1.66	29.04 ± 1.67	29.97 ± 2.24	29.4 ± 2.56	29.94 ± 2.36	29.56 ± 1.66
	**0.5**	30.01 ± 0.95	29.96 ± 2.27	29.87 ± 1.65	29.05 ± 1.23	29.5 ± 1.73	29.94 ± 1.58
	**0.6**	29.64 ± 2.25	30.07 ± 0.93	29.81 ± 2.35	29.68 ± 1.53	29.8 ± 0.90	29.65 ± 2.08
	**0.7**	29.95 ± 1.68	29.83 ± 1.25	29.67 ± 1.52	29.35 ± 2.24	29.6 ± 1.82	29.24 ± 1.85

Table S7. Comparison of Ct values between the national reference method and RT–qPCR methods

Number	RT-qPCR (CT)				National reference method (CT)	
	FAM	ROX	HEX	CY5	FAM	HEX
1	25	N	N	N	25.01	N
2	27.35	N	N	N	24.46	N
3	N	N	N	N	N	N
4	20.8	N	N	N	20.74	N
5	N	N	N	N	N	N
6	27.99	N	N	N	25.66	N
7	N	N	N	N	N	N
8	N	N	N	N	N	N
9	N	N	N	N	N	N
10	N	N	N	N	N	N
11	N	N	N	N	N	N
12	17.53	N	N	N	19.31	N
13	16.58	N	N	17.86	18.5	N
14	N	N	N	N	N	N
15	N	N	N	N	N	N
16	N	N	N	N	N	N
17	24.78	N	N	N	27.15	N

18	18.56	N	N	N	19.89	N
19	23.41	N	N	N	23.55	N
20	22.36	N	N	N	24.17	N
21	16.07	N	N	N	17.07	N
22	17.05	N	N	N	17.89	N
23	21.95	N	N	N	22.43	N
24	26.31	N	N	N	26.48	N
25	34.21	N	N	N	32.34	N
26	N	N	N	N	N	N
27	N	N	N	N	N	N
28	N	N	N	N	N	N
29	27.1	N	N	27.33	28.31	N
30	35.25	N	37.67	N	37.45	38.98
31	N	N	N	N	N	N
32	N	N	N	N	N	N
33	N	N	N	N	N	N
34	N	N	N	N	N	N
35	N	N	N	N	31.15	N
36	26.47	N	N	N	27.73	N
37	29.88	N	N	N	28.99	N
38	N	N	N	N	N	N
39	18.53	N	21.92	N	20.21	22.03
40	N	N	N	N	N	N
41	N	N	N	N	N	N
42	N	N	N	N	N	N
43	N	N	N	N	N	N
44	N	N	N	N	N	N
45	27.87	N	N	N	N	N
46	N	N	N	N	N	N
47	N	N	N	N	N	N
48	N	N	N	N	N	N
49	N	N	N	N	N	N
50	N	N	N	N	N	N
51	N	N	N	N	N	N
52	N	N	N	N	N	N
53	N	N	N	N	N	N
54	N	N	N	N	N	N
55	N	N	N	N	N	N
56	N	N	N	N	N	N
57	N	N	N	N	N	N
58	N	N	N	N	N	N
59	N	N	N	N	N	N
60	N	N	N	N	N	N
61	N	N	N	N	N	N
62	N	N	N	N	N	N
63	N	N	N	N	N	N
64	N	N	N	N	N	N
65	N	N	N	N	N	N
66	N	N	N	N	N	N
67	N	N	N	N	N	N
68	N	N	N	N	33.46	N

69	N	N	N	N	N	N
70	N	N	N	N	N	N
71	N	N	N	N	N	N
72	N	N	N	N	N	N
73	N	N	N	N	N	N
74	N	N	N	N	N	N
75	N	N	N	N	N	N
76	N	N	N	N	N	N
77	N	N	N	N	N	N
78	N	N	N	N	N	N
79	N	N	N	N	N	N
80	N	N	N	N	N	N
81	N	N	N	N	N	N
82	N	N	N	N	N	N
83	N	N	N	N	N	N
84	N	N	N	N	N	N
85	N	N	N	N	N	N
86	N	N	N	N	N	N
87	N	N	N	N	N	N
88	N	N	N	N	N	N
89	N	N	N	N	N	N
90	N	N	N	N	N	N
91	N	N	N	N	N	N
92	N	N	N	N	N	N
93	N	N	N	N	N	N
94	27.54	N	N	N	29.68	N
95	31.79	N	N	N	32.03	N
96	N	N	N	N	N	N
97	N	N	N	N	N	N
98	27.03	N	31.6	N	27.19	30.23
99	27.22	N	31.35	N	27.44	29.56
100	24.09	N	28.16	N	25.34	27.65
Positive control	25.45	N	26.01	N	25.73	26.65
Negative control	N	N	N	N	N	N
